# Smurf1‐targeting microRNA‐136‐5p‐modified bone marrow mesenchymal stem cells combined with 3D‐printed β‐tricalcium phosphate scaffolds strengthen osteogenic activity and alleviate bone defects

**DOI:** 10.1002/kjm2.12847

**Published:** 2024-05-31

**Authors:** Gang Duan, Ya‐Fei Lu, Hong‐Liang Chen, Zi‐Qiang Zhu, Shuo Yang, Yun‐Qing Wang, Jian‐Qiang Wang, Xing‐Hai Jia

**Affiliations:** ^1^ Department of Orthopedics The Second Affiliated Hospital of Xuzhou Medical University Xuzhou Jiangsu China; ^2^ Graduate School of Xuzhou Medical University Xuzhou Jiangsu China; ^3^ Department of Orthopedics The Affiliated Hospital of Xuzhou Medical University Xuzhou Jiangsu China

**Keywords:** BMSCs, bone defect, miR‐136‐5p, Smurf1, β‐TCP

## Abstract

Suitable biomaterials with seed cells have promising potential to repair bone defects. However, bone marrow mesenchymal stem cells (BMSCs), one of the most common seed cells used in tissue engineering, cannot differentiate efficiently and accurately into functional osteoblasts. In view of this, a new tissue engineering technique combined with BMSCs and scaffolds is a major task for bone defect repair. Lentiviruses interfering with miR‐136‐5p or Smurf1 expression were transfected into BMSCs. The effects of miR‐136‐5p or Smurf1 on the osteogenic differentiation (OD) of BMSCs were evaluated by measuring alkaline phosphatase activity and calcium deposition. Then, the targeting relationship between miR‐136‐5p and Smurf1 was verified by bioinformatics website analysis and dual luciferase reporter assay. Then, a rabbit femoral condyle bone defect model was established. miR‐136‐5p/BMSCs/β‐TCP scaffold was implanted into the defect, and the repair of the bone defect was detected by Micro‐CT and HE staining. Elevating miR‐136‐5p‐3p or suppressing Smurf1 could stimulate OD of BMSCs. miR‐136‐5p negatively regulated Smurf1 expression. Overexpressing Smurf1 reduced the promoting effect of miR‐136‐5p on the OD of BMSCs. miR‐136‐5p/BMSCs/β‐TCP could strengthen bone density in the defected area and accelerate bone repair. SmurF1‐targeting miR‐136‐5p‐modified BMSCs combined with 3D‐printed β‐TCP scaffolds can strengthen osteogenic activity and alleviate bone defects.

## 1 INTRODUCTION

Bone defect is a common clinical‐pathological phenomenon, mostly appearing in patients with tumor resection, sports injury, and infection as well as patients with initial joint replacement and revision, which may cause serious damage to the normal musculoskeletal system and psychology of the human body, and usually requires surgical bone grafting to repair the lesions.^1^, ^2^ However, due to the shortcomings of clinical transplantation materials, the repair of bone defects presents a complex process, so the reconstruction of bone shape and function is still faced with great challenges, especially the repair of large‐volume bone defects, which is the difficulty of clinical treatment at present. Therefore, the search for the repair method for bone defects has been the subject of continuous in‐depth research.

3D bioprinting technique can directly, accurately, and quickly design and produce personalized materials with high precision, complex structure, and certain biological activity by using appropriate bioprinters and materials under the control of computer software and preset data.^3^, ^4^ β‐c (β‐TCP) has a chemical structure similar to natural bone^5^ and is the most promising biological material due to its bone transduction, immune response deficiencies, and unlimited availability.^6^, ^7^ However, β‐TCP faces a challenge in being applied in bone defect repair because of the lack of osteogenic induction properties.

Tissue engineering is a perspective method to repair bone defects by a combination of biomaterial science, cell biology, and biomedical engineering.^8^, ^9^ Significantly, modifying osteogenic genes in bone marrow mesenchymal stem cells (BMSCs) has become a promising therapeutic approach. Currently, functional gene molecules such as DNA plasmid,^10^, ^11^ siRNA,^12^ and miRNA^13^ have been employed to bone tissue engineering. In contrast to pDNA method,^14^ ncRNAs can modify gene expression when delivered to the cytoplasm. Furthermore, ncRNAs as a bioactive factor in bone tissue engineering have become a new star in bone defect repair.^15^

Ubiquitination can translate and modify proteins as well as degrade endogenous proteins.^16^ As the most concerned ubiquitin enzyme in bone biology,^17^ Smurf1 is a key mediator in bone formation. For example, Smurf1 mediates Runx2 degradation and inhibits osteoblast differentiation and bone formation.^18^ Inhibition of Smurf1 expression can stimulate osteogenic differentiation (OD) of MSCs and bone formation.^19^ Smurf1 has also been considered to mediate Smad1/5 degradation, thus inhibiting osteoblast differentiation.^20^, ^21^ This study found that miR‐136‐5p may be a miRNA regulating Smurf1 through the bioinformatics website starBase. Therefore, this study aims to clarify the mechanism by which miR‐136‐5p inhibits Smurf1 expression, providing a bone tissue engineering method through miR‐136‐5p‐modified BMSCs with 3D printed β‐TCP scaffolds.

## 2 METHODS

### 2.1 Preparation of 3D printed β‐TCP scaffolds

Commercial β‐TCP scaffolds (average particle size: 550 nm) were purchased from Berkeley Advanced Biomaterials (CA, USA). The scaffolds (7 mm in diameter and 10 mm in height) were developed in a CAD file and fed into a 3D printer (ProMetal, USA). The 500 μm connected holes were square and distributed in X, Y, and Z directions. In addition to the optimized layer thickness of 20 μm, 3D printing process parameters such as drying time, binder drop volume, and saturation may be optimized.^22^ The scaffolds were treated by a blast blower, solidified at 175°C for 1.5 h, sintered at 1250°C by a muffle furnace for 2 h, and cooled to room temperature.

### 2.2 Characteristics of β‐TCP scaffolds

The compression strength of the β‐TCP scaffolds was evaluated using a universal testing machine (AG‐IS, Shimadzu, Japan) with a constant crosshead velocity of 0.33 mm/min. The compression strength is calculated from the maximum bearing load and the size of the sintered support. The scaffolds were observed by electric field emission scanning electron microscope (FEI Inc., OR, USA). The porosity of the scaffolds was calculated using the ethanol replacement method,^23^ according to the following formula: Porosity (%) = (V1 − V3)/(V2 − V3) × 100%, where V1 is the total volume after adding absolute ethanol to the graduated cylinder and immersing the scaffold sample in it. V2 is the total volume recorded after the air in the pores of the material was evacuated by a vacuum desiccator, and the pores were filled with ethanol. V3 is the total volume of the material after the graduated cylinder was removed.

### 2.3 Collection, culture, and validation of rabbit BMSCs

BMSCs were isolated from rabbit tibia and femur by density gradient centrifugation (800 *g*, 5 min, 22°C), and those with adherence were screened.^24^ BMSCs were cultured in low glucose‐DMEM supplemented with 10% FBS and 1% penicillin–streptomycin at 5% CO_2_ at 37°C.

Cells were plated in 12‐well plates (5 × 10^4^ cells/well). Single‐cell suspensions were packed in Eppendorf (EP) tubes, each containing 200 μL cell suspension (5 × 10^6^ cells/mL). CD29 (FITC‐conjugated), CD34 (FITC‐conjugated), and CD44 (phycoerythrin‐conjugated) antibodies for flow cytometry and FITC‐labeled IgG were added to the EP tube. All antibodies (Biosciences, NJ, USA) were incubated at 4°C for 1 h, washed three times with PBS containing 3% FBS, centrifuged at 4°C 120 *g* for 5 min and then resuspended at 4°C for 1 h with 200 μL polyoxymethyl (4%). The cell phenotypes were analyzed by flow cytometry (FACS Gallios, Beckman, USA).

### 2.4 Construction and transduction of lentiviral vectors

miR‐NC, miR‐136‐5p, sh‐NC, sh‐Smurf1, oe‐NC, and oe‐Smurf1 (GenePharma, Shanghai, China) were transfected into BMSCs. After 48 h, the supernatant was collected and filtered and concentrated using the Centricon Plus‐20 filter (Millipore, MA, USA). For lentivirus transduction, the medium was replaced with Opti‐MEM (Invitrogen), and 5 mg/mL polybrene (GeneChem) was added, followed by concentrated viral supernatant (8 μg/mL). miR‐136‐5p and Smurf1 after 48 h were detected by RT‐qPCR to verify the success of lentivirus transfection.

### 2.5 BMSC grouping

BMSCs were divided into the following seven groups: BMSCs group (without transfection); miR‐NC/BMSCs group (transfection with miR‐NC); miR‐136‐5p/BMSCs group (transfection with miR‐136‐5p); sh‐NC/BMSCs group (transfection with sh‐NC); sh‐Smurf1/BMSCs group (transfection with sh‐Smurf1); miR‐136‐5p + oe‐NC/BMSCs group (transfection with miR‐136‐5p mimic + oe‐NC); miR‐136‐5p + oe‐Smurf1/BMSCs group (transfection with miR‐136‐5p mimic + oe‐Smurf1).

### 2.6 Induction of osteoblast differentiation

BMSCs were plated into 6‐well plates (1 × 10^5^ cells/well) and cultured in an osteoblast‐specific induction medium containing 0.05 mM ascorbic acid, 10 mM β‐glycerophosphate, and 100 nM dexamethasone (Cyagen, China) to induce OD. The medium was changed every 2 days for 14 days. The cells were then collected and analyzed on a specified day.

### 2.7 RT‐qPCR

TRIzol reagent (Invitrogen; Thermo Fisher Scientific) was employed for total RNA extraction. cDNA was obtained through reverse transcription from 2 μg RNA using a Biochemical kit (Takara, Japan), amplified using SYBR GREEN PCR Master mix (Takara), and analyzed in the ABI7500 Real‐time PCR system (Applied Biosystems; Thermo Fisher Scientific). Data were analyzed by 2^−ΔΔCt^. miR‐136‐5p, and Smurf1 levels were normalized to U6 and GAPDH, respectively. The cycle conditions of qPCR were: predenaturation at 95°C for 10 min, denaturation at 95°C for 10 s, annealing at 58°C for 20 s, and extension at 72°C for 10 s, 40 cycles in total. Table 1 presents the primer sequences.

**TABLE 1:** Primers.

Genes	Primers (5′–3′)
miR‐136‐5p	F: GCGACTCCATTTGTTTTGA
	R: GCAGGGTCCGAGGTATTC
Smurf1	F: AGTTCGTGGCCAAATAGTGG
	R: GTTCCTTCGTTCTCCAGCAG
Runx2	F: GCCTTCAAGGTTGTAGCCCT
	R: TGAACCTGGCCACTTGGTTT
OCN	F: TGAGAGCCCTCACACTCCTC
	R: CGCCTGGGTCTCTTCACTAC
U6	F: CTCGCTTCGGCAGCACA
	R: AACGCTTCACGAATTTGCGT
GAPDH	F: CGGAGTCAACGGATTTGGTCGTAT
	R: AGCCTTCTCCATGGTGGTGAAGAC

Abbreviations: GAPDH, Glyceraldehyde‐3‐phosphate dehydrogenase; miR‐136‐5p, microRNA‐136‐5p; OCN, osteocalcin; Runx2, Runt‐related transcription factor 2; Smurf1, Smad ubiquitylation regulatory factor‐1.

### 2.8 Western blot

Total proteins were extracted by radioimmunoprecipitation test buffer (Sigma‐Aldrich) containing a protease inhibitor (Thermo Fisher Scientific) and quantified using the protein detection kit (Qcbio Science Technologies, Shanghai, China). The total protein (30 μg) was separated by 10% SDS‐PAGE electrophoresis and transferred to a PVFD (Millipore, USA) membrane, which was then sealed with 5% skim milk and mixed with primary antibodies Smurf1 (ab236081; Abcam), Runx2 (ab76956; Abcam), OCN (ab13420; Abcam) or GAPDH (1:2000; Santa Cruz Biotechnique) overnight at 4°C. The membrane was cleaned and incubated with a secondary antibody (1:5000) coupled with horseradish peroxidase for 2 h. ECL assay was performed using the ECL substrate kit (Thermo Fisher Scientific).

### 2.9 Alkaline phosphatase activity determination

Seven days after osteogenic induction, cells were fixed with 4% paraformaldehyde for 30 min and washed with PBS three times. Alkaline phosphatase (ALP) staining was performed using the ALP kit (Beyotime, China) and observed under an optical microscope (Olympus, Japan).

### 2.10 Calcium deposition determination

After 14 days of osteogenic induction, cells were stained with 0.2% Alizarin Red S staining solution (Sigma‐Aldrich; Merck KGaA), rinsed with PBS, and observed under an optical microscope (Olympus).

### 2.11 Luciferase activity detection

starBase predicted potential miR‐136‐5p binding sites in Smurf1 3′UTR. Sequences containing Smurf1 wild type (Smurf1‐WT) or mutant type (Smurf1‐mut) seed regions were synthesized and cloned into luciferase reporter plasmids. BMSCs were co‐transfected with miR‐136‐5p or miR‐NC. After 24 h, luciferase activity was measured using Dual luciferase Assay Kit (Promega, USA).

### 2.12 Formation of BMSCs/β‐TCP scaffolds

The average pore size of β‐TCP scaffolds is 360 μm ± 40 μm, with a porosity of 65%. BMSCs (1.0 × 10^7^ cells/mL) containing lentivirus transduction of miR‐NC, miR‐136‐5p, oe‐NC, and oe‐Smurf1 were added to saturate β‐TCP. BMSCs were incubated with β‐TCP for at least 4 h.

### 2.13 Femur defect model

Animal treatments were conducted following the Animal Welfare Act and NIH Guide. New Zealand White rabbits (male, 2.0–2.5 kg, 2–3 months old) were purchased from Laboratory Animal Center of Guangzhou University of Traditional Chinese Medicine (China). Each rabbit was anesthetized by intramuscular injection of pentobarbital sodium (25 mg/kg) and methylbenzene (8 mg/kg) before surgery to relieve pain. The femur of each rabbit was cut and scrubbed with iodophor and 75% ethanol solution. The skin was cut longitudinally on the lateral condyle of the rabbit femur to make a 5 cm incision. Then the skin and muscle tissue were dissected, and the femoral condyle was then exposed. A critical size defect (7 mm in diameter, 10 mm in depth) was established on the femoral condyle using dental burr. The scaffolds were then implanted into the bone defect area. Finally, the muscle and skin was repaired in layers. After surgery, all rabbits received intramuscular injections of antibiotics for 3 days. The rabbits were kept freely in cages and fed and watered according to traditional eating habits. Animals were euthanized 3 months after implantation.

### 2.14 Micro‐CT

The newly formed bone around the scaffold was evaluated based on GE eXplore Locus SP Micro‐CT. The bones were scanned by GEHC MicroView software (GE Healthcare BioSciences, UK) at 80 kV and 80 μA, with exposure time of 3000 ms and resolution of 15 μm. Region of interest (ROI) was selected according to the anatomical location of the bone defect. First, the CT value of the new bone tissue was used to identify and select the new bone tissue in the ROI. Then, the scaffold in the ROI was identified and selected by using the CT value of the scaffold. By removing the selected portion of the scaffold from the selected portion of the newly formed bone tissue, the actual portion of the newly formed bone tissue in the scaffold could be obtained. Bone mineral density (BMD), trabecular thickness (TbTh), bone volume fraction (bone volume/total volume [BV/TV]), and trabecular number (TbN) were calculated.

### 2.15 HE staining

After Micro‐CT examination, the femur tissue was thoroughly cleaned with saline, then fixed in 4% paraformaldehyde, followed by decalcification in 10% EDTA (PH 7.0) for 5 weeks. After dehydration and complete decalcification, the sample was embedded in paraffin wax and prepared into 5 μm vertical continuous sections. HE staining was then conducted, and images were observed by an optical microscope (Olympus).

### 2.16 Statistical analysis

All data were expressed as mean ± standard deviation and compared by unpaired Student's *t* test. GraphPad Prism software (version 8.1.2) was employed for all statistical analyses, with a statistical significance of *p* < 0.05.

## 3 RESULTS

### 3.1 Characterization of a 3D printed β‐TCP scaffold

**FIGURE 1:**
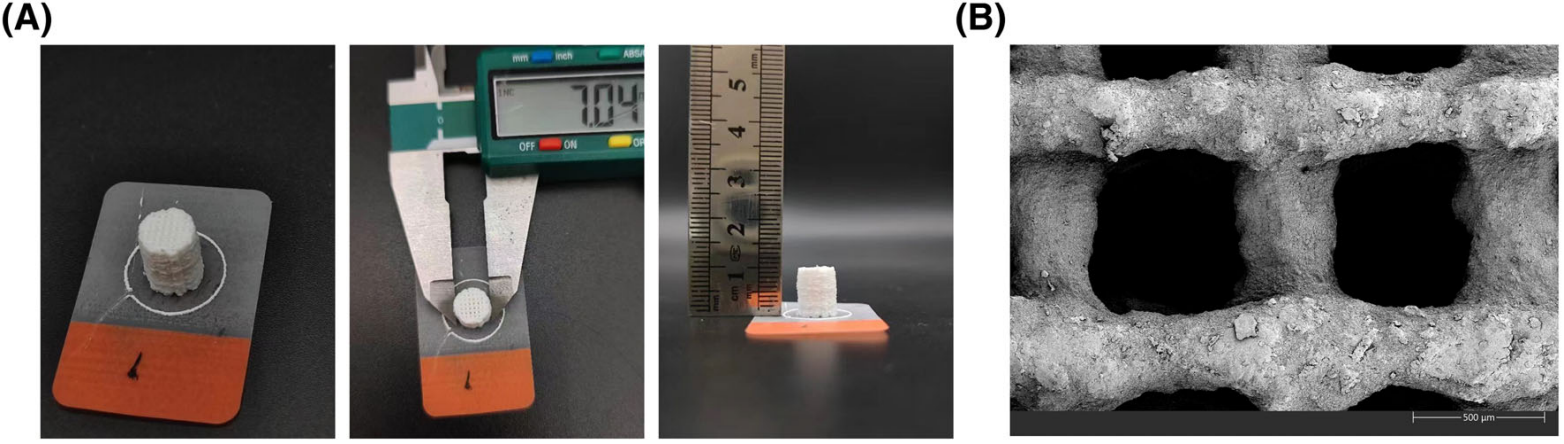
Characterization of 3D printed β‐TCP scaffold. (A) The mean diameter and height of the β‐TCP scaffold. (B) SEM images of the surface topography of β‐TCP scaffold.

The 3D‐printed β‐TCP scaffolds were made from CAD files using a 3D printer, and were designed to have porous channels from the top to the sides of each scaffold, forming a lattice network through interconnected pores. The 3D printed β‐TCP scaffolds were cylindrical, with an average diameter of 7 mm and an average height of 10 mm, white in color, with a rough surface and uniform texture (Figure 1A). The microstructure of the PCL/β‐TCP/CS composite scaffold is shown in Figure 1B. The β‐TCP scaffolds were filled with interlocking pore structures inside, the pores were interconnected, and the average diameter of the micropores was 360 μm ± 40 μm. In addition, the porosity of β‐TCP scaffold was 65%, the density was 0.87 ± 0.03 g/cm^3^, and the compression strength was 5.48 ± 0.04 MPa.

### 3.2 Characteristics of rabbit BMSCs

**FIGURE 2:**
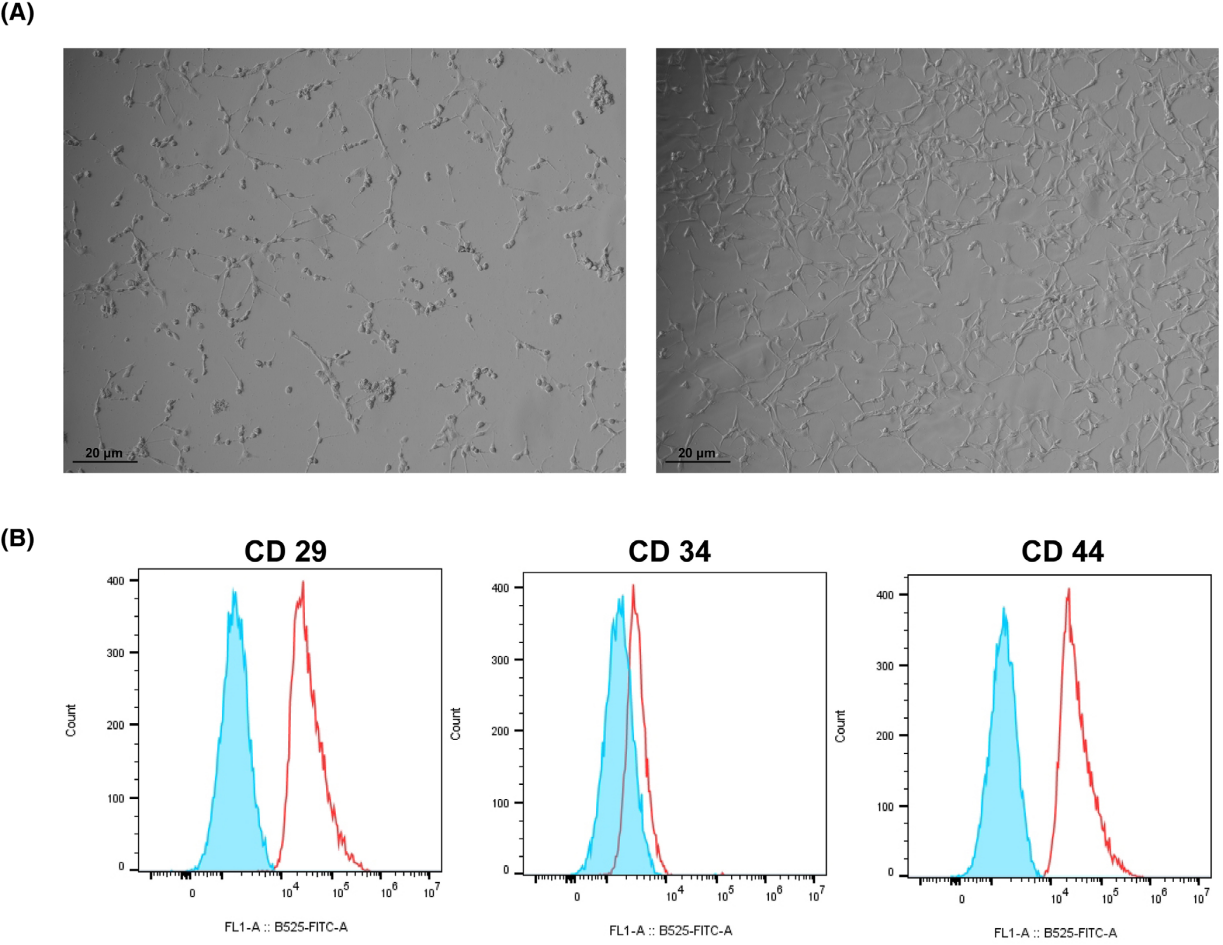
Characteristics of rabbit bone marrow mesenchymal stem cells (BMSCs). (A) The morphology of rabbit BMSCs. (B) Flow cytometry analysis of cell surface markers.

Rabbit BMSCs were rounded at passage 1 and fusiform at passage 3 (Figure 2A). BMSC surface markers detected by flow cytometry showed positive expressions of CD29 and CD44, while negative expressions of CD34 (Figure 2B). It was proved that the cultured cells were rabbit BMSCs, which could be used for follow‐up experiments.

### 3.3 Upregulating miR‐136‐5p or downregulating Smurf1 can stimulate OD of BMSCs

**FIGURE 3:**
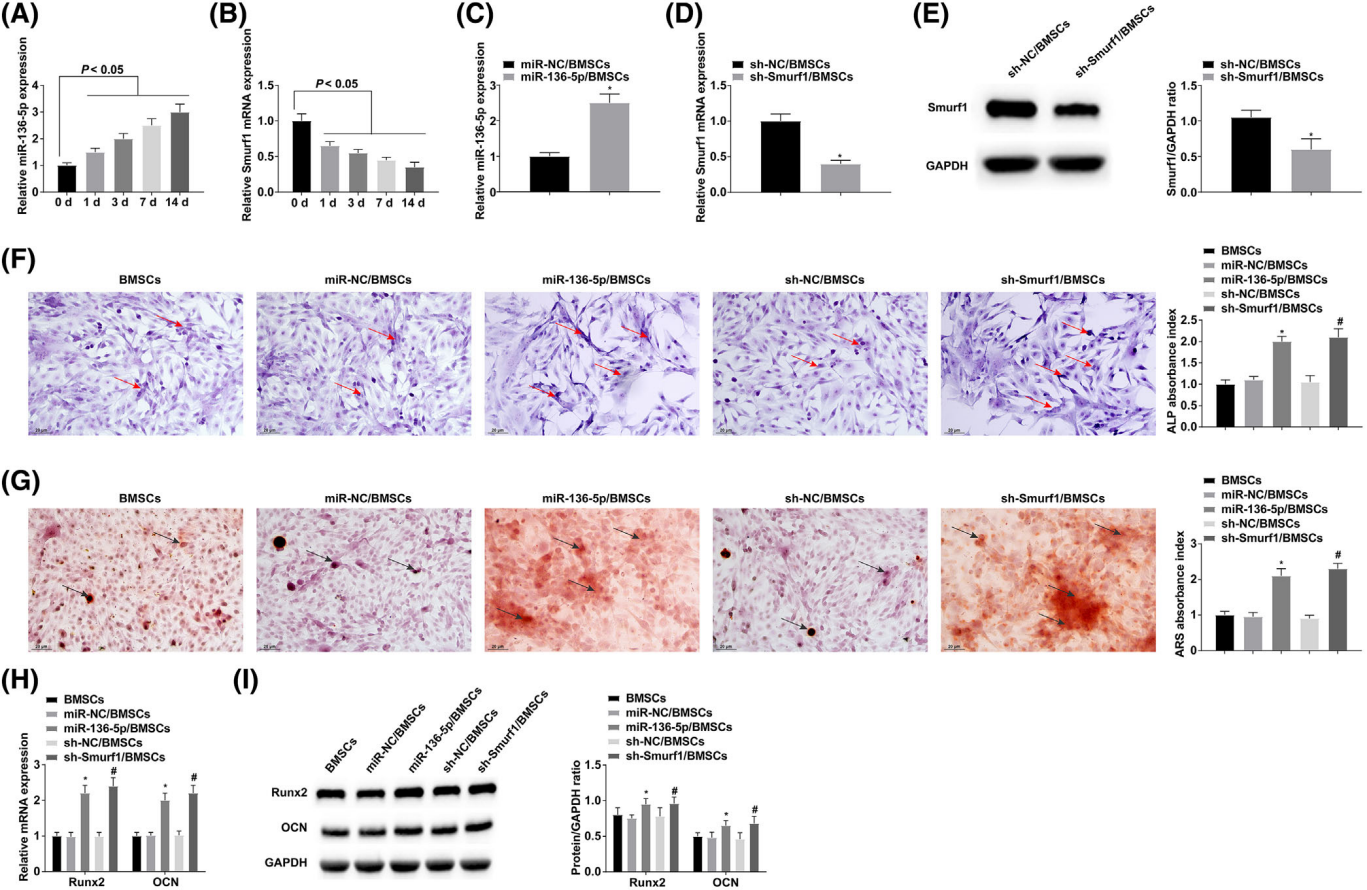
Up‐regulating miR‐136‐5p or down‐regulating Smurf1 can stimulate OD of bone marrow mesenchymal stem cells (BMSCs). (A and B) RT‐qPCR tested miR‐136‐5p and Smurf1 during OD of BMSCs. (C–E) RT‐qPCR or Western blot verified successful transfection. (F) ALP staining, arrows indicate ALP‐positively stained cells. (G) Alizarin red staining, arrows indicate alizarin red positively stained cells. (H and I) RT‐qPCR and Western blot analysis of Runx2 and OCN. *p < 0.05 versus miR‐NC/BMSCs; ^#^ *p* < 0.05 versus sh‐NC/BMSCs.

RT‐qPCR results showed that miR‐136‐5p was upregulated and that of Smurf1 was downregulated during BMSC OD (Figure 3A,B). To further explore their actions in OD, miR‐NC, miR‐136‐5p, sh‐NC, or sh‐Smurf1 were transfected into BMSCs (Figure 3C–E). ALP is an early marker of osteoblast differentiation, and its activity reflects osteoblast maturation. At the same time, alizarin red staining was used to detect calcium deposition on the cell surface to verify the successful differentiation of BMSCs into osteoblasts. ALP and alizarin red staining results showed that ALP activity and calcium deposition were increased after overexpressing miR‐136‐5p or reducing Smurf1 (Figure 3F,G). Osteogenic genes Runx2 and OCN were detected, and results manifested that overexpressing miR‐136‐5p or silencing Smurf1 significantly promoted their expression levels (Figure 3H,I).

### 3.4 miR‐136‐5p negatively regulates Smurf1 expression

**FIGURE 4:**
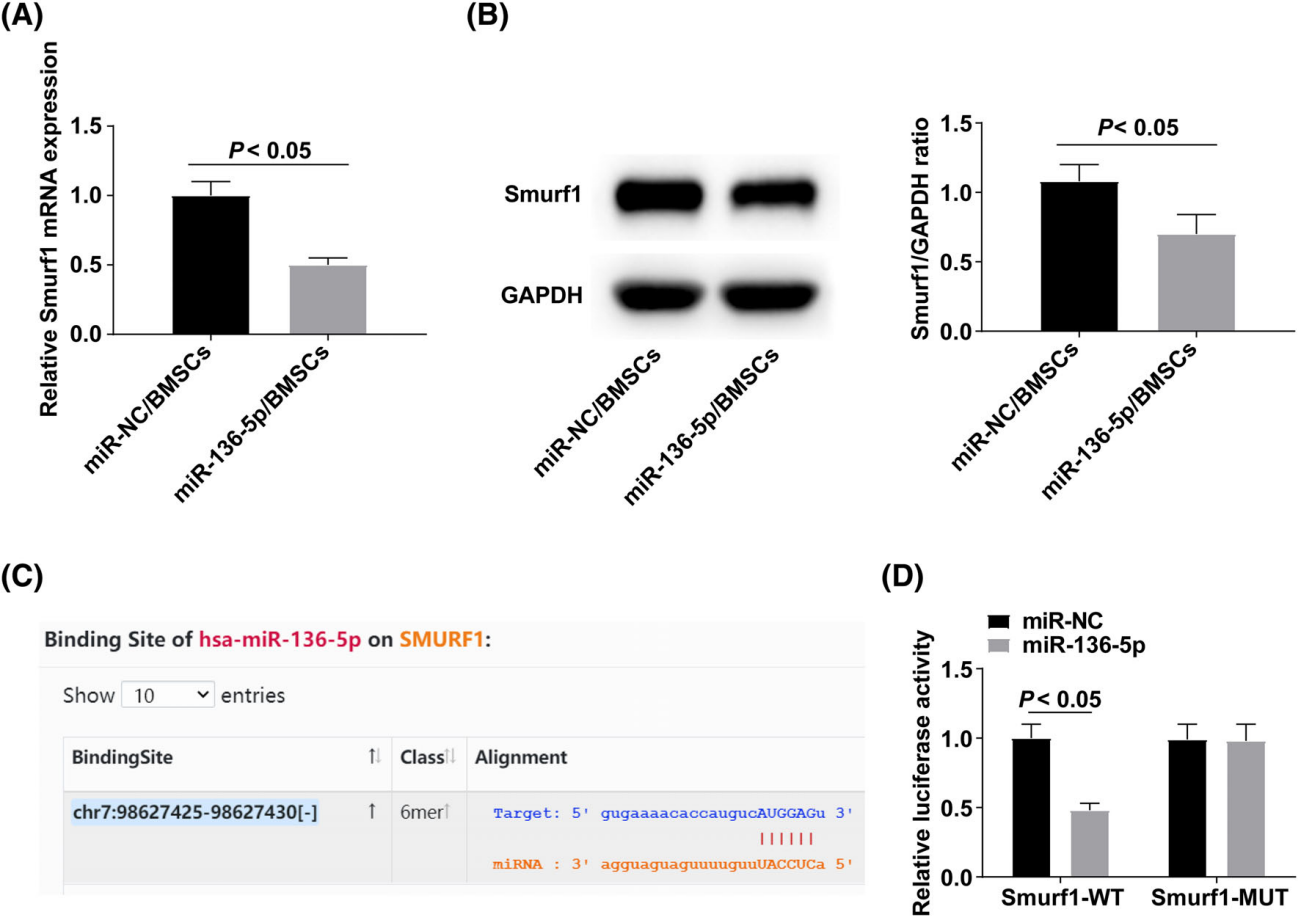
Smurf1 expression is negatively regulated by targeting miR‐136‐5p. (A and B) RT‐qPCR and Western blot analysis of Smurf1 after up‐regulation of miR‐136‐5p. (C) Bioinformatics website starBase found the presence of targeted binding sites between miR‐136‐5p and Smurf1. (D) Dual luciferase reporter assay to verify the targeting relationship between miR‐136‐5p and Smurf1.

Smurf1 after the up‐regulation of miR‐136‐5p was evaluated by RT‐qPCR and Western blot. The results showed that, after upregulating miR‐136‐5p, Smurf1 expression was significantly decreased (Figure 4A,B). starBase found that there was a targeted binding site between miR‐136‐5p and Smurf1 (Figure 4C), and dual luciferase reporter assay results showed that after co‐transfection of Smurf1‐WT and miR‐136‐5p, the luciferase activity significantly decreased (Figure 4D).

### 3.5 Promoting Smurf1 expression can weaken the impact of miR‐136‐5p up‐regulation on OD of BMSCs

**FIGURE 5:**
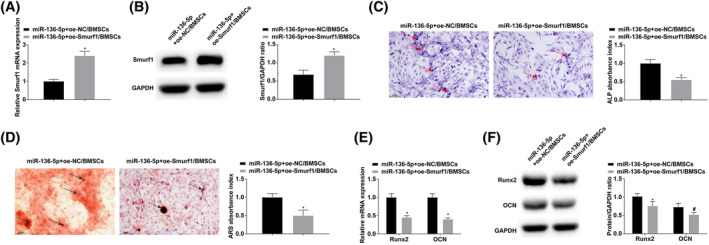
Promoting Smurf1 expression can weaken the effect of miR‐136‐5p up‐regulation on OD of bone marrow mesenchymal stem cells. (A and B) RT‐qPCR and Western blot analysis of Smurf1. (C) ALP staining, arrows indicate ALP‐positively stained cells. (D) Alizarin red staining, arrows indicate alizarin red positively stained cells. (E and F) RT‐qPCR and Western blot analysis of Runx2 and OCN. *p < 0.05 versus miR‐136‐5p + oe‐NC.

miR‐136‐5p + oe‐NC or miR‐136‐5p + oe‐Smurf1 were transfected into BMSCs (Figure 5A,B). Experimental results showed that inducing Smurf1 could impair the impact of miR‐136‐5p up‐regulation on the OD of BMSCs (Figure 5C–F).

### 3.6 miR‐136‐5p/BMSCs/β‐TCP can strengthen bone density and accelerate bone defect repair

**FIGURE 6:**
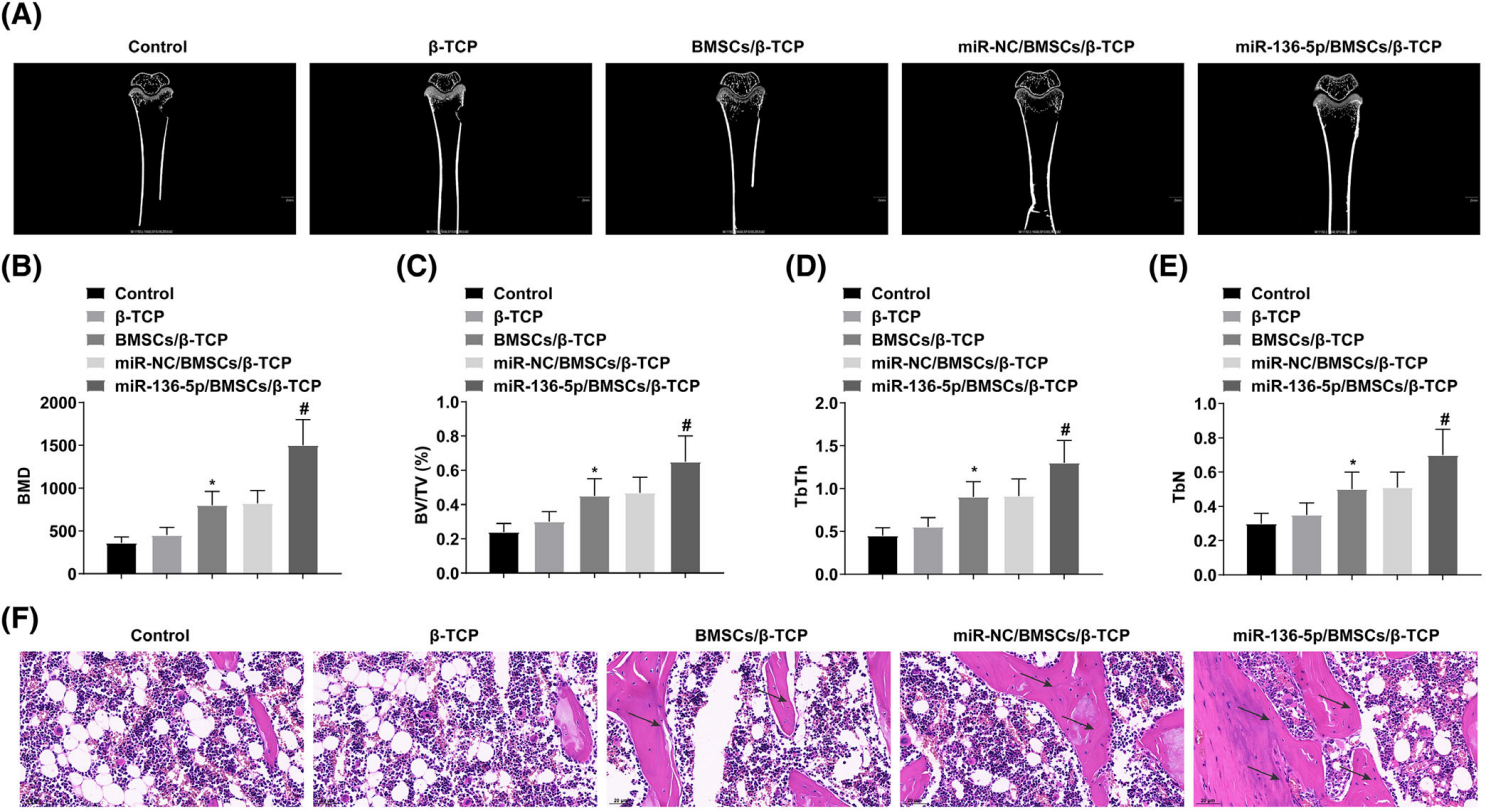
miR‐136‐5p/BMSCs/β‐TCP can strengthen bone density in the defect area and accelerate bone defect repair. (A) Micro‐CT analysis of new bone formation. (B–E) Quantitative analysis of BMD, BV/TV, TbTh, and TbN levels. (F) HE staining, arrows indicate new bone formation. *p < 0.05 versus β‐TCP; ^#^ *p* < 0.05 versus miR‐NC/BMSCs/β‐TCP.

A rabbit femur defect model was established. BMSCs containing lentivirus transduction of miR‐NC or miR‐136‐5p were loaded onto β‐TCP scaffolds, and then implanted into the femoral defect area of rabbit. Micro‐CT analysis of femur samples showed that no significant new bone formation was observed in the Control group and the β‐TCP group, but new bone formation was observed in the BMSCs/β‐TCP group, miR‐NC/BMSCs/β‐TCP group, and miR‐136‐5p/BMSCs/β‐TCP group. miR‐136‐5p/BMSCs/β‐TCP group completed bone defect repair (Figure 6A). It should be considered that the scaffold material was mostly degraded after 3 months so it cannot be shown by micro‐CT scans. This phenomenon suggests that miR‐136‐5p‐modified BMSCs combined with β‐TCP scaffolds accelerate bone defect repair. Quantitative analysis showed that BMD, BV/TV, TbTh, and TbN levels in miR‐136‐5p/BMSCs/β‐TCP group were highest (Figure 6B–E). To confirm the Micro‐CT findings, HE staining was performed on the femur tissue sections. miR‐136‐5p/BMSCs/β‐TCP group had repaired most of the bone defect areas, but in other groups, there were a large number of unrepaired areas, and repair degree was relatively low (Figure 6F).

### 3.7 miR‐136‐5p‐modified BMSCs targeting Smurf1 combined with β‐TCP strengthen bone density in the defect area and accelerate bone defect repair

**FIGURE 7:**
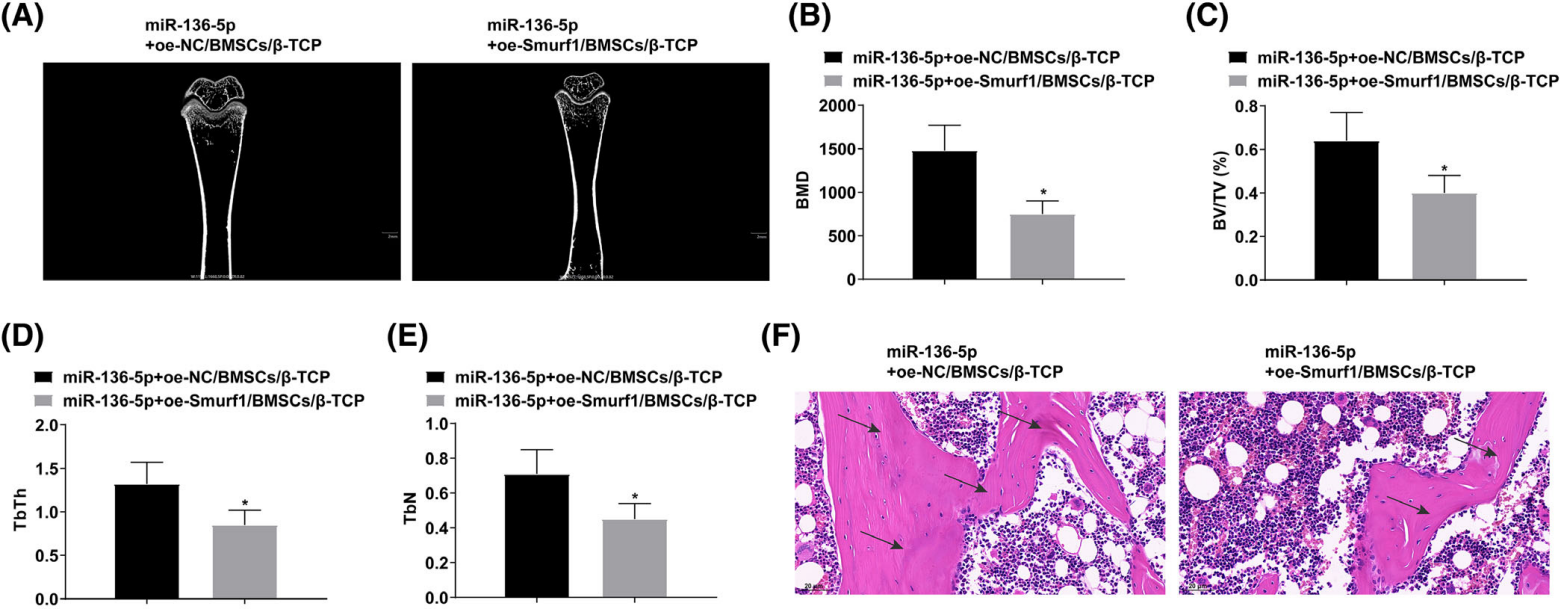
miR‐136‐5p/BMSCs/β‐TCP can strengthen bone density in the defect area and accelerate bone defect repair. (A) Micro‐CT analysis of new bone formation. (B–E) Quantitative analysis of BMD, BV/TV, TbTh, and TbN levels. (F) HE staining, arrows indicate new bone formation. *p < 0.05 versus miR‐136‐5p + oe‐NC/BMSCs/β‐TCP.

The bone defect repair effect of miR‐136‐5p + oe‐Smurf1/BMSCs/β‐TCP group was significantly lower than that of miR‐136‐5p + oe‐NC/BMSCs/β‐TCP group (Figure 7A–F). This phenomenon suggests that miR‐136‐5p/BMSCs can accelerate bone repair by targeting Smurf1.

## 4 DISCUSSION

According to the type and severity of bone defect, the repair methods vary greatly. Autologous bone is widely recognized as the best replacement material in bone transplantation, with the effects of bone induction, conduction, and genesis. After transplantation, the activities of osteoblasts can be maintained to a large extent. Meanwhile, the bone matrix contains a variety of bioactive substances, which can accelerate the differentiation of osteoblasts, and rapidly integrate with the surrounding bone tissue for growth.^25^ However, it is limited by insufficient factors such as limited supply, extended trauma, increased secondary operation, and spread of infection.^26^ The clinical application of allograft bone is also limited due to the possibility of immune rejection and disease transmission, as well as economic and ethical reasons.^1^ Therefore, it is necessary to seek a new method of bone defect repair.

Materials that mimic the functional and structural features of natural tissues remain a challenge. Over the past few decades, bioceramics have emerged as viable candidates for orthopedic, maxillofacial, and dental applications.^27^ Calcium phosphate materials, especially β‐TCP, have been widely used in non‐low‐load orthopedic implants due to their similar composition to natural bone, inherent bone conductivity, and customizable biodegradability.^28^, ^29^ However, β‐TCP is limited by its inherent osteogenic deficiency.^30^, ^31^ The three main elements of tissue engineering are seed cells, scaffold materials, and growth factors. Finding suitable seed cells is an important factor in tissue engineering. BMSCs are stem cells with multi‐differentiation potential in bone marrow, and can differentiate into various cell lines under specific induction conditions.^24^ BMSCs have become ideal seed cells for tissue engineering, cell engineering, and gene engineering due to their advantages such as convenient sampling, easy culture and amplification, and weak immunogenicity.^32^ Tissue engineering methods using BMSCs have attracted people's attention in the reconstruction of large bone defects.^33^ Several studies have demonstrated the osteogenic ability of implants prepared with BMSCs to achieve bone defect regeneration.^34^, ^35^ The results showed that BMSCs combined with β‐TCP scaffolds implanted in the defect area of rabbit femur strengthened bone density in the defect area and accelerated bone defect repair.

miR‐136‐3p could strengthen OD of BMSCs by targeting PTEN.^36^ Given the homology of miR‐136‐3p and miR‐136‐5p, we suspect that miR‐136‐5p may regulate bone formation like miR‐136‐3p. Smurf1 negatively regulates osteoblast proliferation, differentiation, and maturation.^37^, ^38^, ^39^ This study noted that upregulating miR‐136‐5p or downregulating Smurf1 stimulated OD of BMSCs, and upregulating Smurf1 weakened the effect of miR‐136‐5p on OD of BMSCs.

Finally, we demonstrated the repair effect of miR‐136‐5p‐modified BMSCs combined with β‐TCP scaffolds in bone defects. The rabbit femoral defect model was established, showing that the miR‐136‐5p/BMSCs/β‐TCP composite scaffold strengthened the bone density in the defect area and induced bone defect repair.

## 5 CONCLUSION

In summary, (1) miR‐136‐5p positively regulates OD of BMSCs based on negatively regulating Smurf1. (2) miR‐136‐5p‐modified BMSCs combined with β‐TCP scaffolds can significantly strengthen bone density and accelerate bone defect repair. (3) A novel miR‐136‐5p/Smurf1 pathway may contribute to OD of BMSCs, thus providing new and deeper insights into the regulatory mechanisms of miR‐136‐5p on OD. (4) Tissue engineering scaffolds combined with miR‐136‐5p‐modified BMSCs and β‐TCP scaffolds are a promising and effective strategy for repairing bone defects.

## CONFLICT OF INTEREST STATEMENT

The authors declare no conflict of interest.

## ETHICS STATEMENT

The present study was approved by the Animal experiments were approved by The Second Affiliated Hospital of Xuzhou Medical University Animal Experimental Ethics Committee. And all procedures complied with the National Institutes of Health Guide for the Use of Laboratory Animals.
